# Clinical characteristics and outcomes of persistent bacteremia in patients with head and neck cancer in a tertiary care hospital

**DOI:** 10.3389/fmed.2024.1406983

**Published:** 2024-06-19

**Authors:** Shiori Kitaya, Hajime Kanamori, Ryo Ishii, Makoto Katsumi, Ryoukichi Ikeda, Kenjiro Higashi, Risako Kakuta, Akira Ohkoshi, Yukio Katori

**Affiliations:** ^1^Department of Infectious Diseases, Internal Medicine, Tohoku University Graduate School of Medicine, Sendai, Japan; ^2^Department of Otolaryngology, Head and Neck Surgery, Tohoku University Graduate School of Medicine, Sendai, Japan; ^3^Department of Laboratory Medicine, Tohoku University Hospital, Sendai, Japan; ^4^Department of Otolaryngology, Head and Neck Surgery, School of Medicine, Iwate Medical University, Shiwa, Iwate, Japan

**Keywords:** catheter-related bloodstream infection, clearance of bacteremia, head and neck cancer, mortality rate, hypoalbuminemia, persistent bacteremia, pyogenic spondylitis

## Abstract

**Introduction:**

Compared to other cancers, research on bloodstream infection in head and neck cancer is scarce, lacking comparative studies on persistent versus transient bacteremia outcomes.

**Methods:**

This retrospective survey examined patients with head and neck cancer undergoing blood culture at our center from June 2009 to May 2023. Blood culture-positive cases suspected of infection were divided into persistent bacteremia and transient bacteremia groups. We investigated their clinical, epidemiological, and microbiological features, including risk factors for persistent bacteremia and mortality. The primary outcome was 90-day mortality.

**Results:**

In this 97-patient cohort, 14 (14%) cases were assigned to the persistent bacteremia group. Catheter-related bloodstream infections were the leading cause of infection in both groups, consistently contributing to a high proportion of overall bloodstream infections. The mortality rate was generally higher in the persistent bacteremia group than in the transient bacteremia group (odds ratio [OR], 2.6; 95% confidence interval [CI], 0.6–11.1), particularly in the non-clearance subgroup (OR, 9; 95% CI, 0.5–155.2). Pyogenic spondylitis was a key risk factor for persistent bacteremia, while hypoalbuminemia increased mortality.

**Conclusion:**

In patients with bacteremia and head and neck cancer, persistent bacteremia was associated with higher mortality than was transient bacteremia. Adittionally, bacteremia clearance in persistent bacteremia is thus crucial for prognostic improvement.

## 1 Introduction

Head and neck cancer (HNC) is the fifth most common malignant tumor in the world and the eighth leading cause of carcinoma-related mortality (1, 2). Chemoradiotherapy (CRT), a common treatment for HNC, can lead to skin and pharyngeal mucosal damage, neutropenia, and immunosuppression (3). Specifically, it can lead to an increased risk of infectious complications, including bloodstream infections (BSIs), both during and after radiotherapy (RT) or CRT. Notably, patients undergoing RT or CRT experience 90-and 180-day overall mortality rates of 3–4% and 7–10%, respectively (4–7), with BSI implicated in 10% of non-carcinoma-related early deaths (8).

Persistent bacteremia (PB) is associated with adverse clinical outcomes, such as prolonged hospitalization and higher mortality rates (9). It is attributed to various organisms, including *Staphylococcus aureus* (10), gram-negative rods (GNR) (11), and *Candida* spp. (12). Furthermore, our previous investigation revealed that for gram-positive cocci (GPC), GNR, and *Candida* spp., the non-clearance group of PB had a higher mortality rate than did the clearance group (13). Although studies on BSI in patients with HNC have been conducted (8, 14–16), none have compared the outcomes between PB and transient bacteremia (TB) in BSI among patients with HNC, nor have they compared outcomes based on clearance of bacteremia. Accordingly, the objectives of this retrospective observational study were to (1) compare clinical outcomes, including mortality rates, between patients with HNC having PB versus TB, (2) compare clinical outcomes based on clearance of bacteremia, and (3) analyze the risk factors leading to PB and mortality in patients with HNC.

## 2 Materials and methods

### 2.1 Study design and setting

This retrospective, single-center, observational study was conducted at a tertiary care center at Tohoku University Hospital, Sendai, Miyagi, Japan. We investigated electronic clinical charts and hospital records to gather research variables from patients with HNC who underwent blood culture (BC) at the Department of Otolaryngology, Head and Neck Surgery, Tohoku University Hospital, between June 2009 and May 2023. All patients with HNC diagnosed with BSI were eligible for inclusion in this study. Exclusion criteria included possible contaminants, such as coagulase-negative staphylococci, *Propionibacterium* spp., and *Corynebacterium* spp.

The clinical characteristics were retrospectively examined using electronic clinical charts, hospital records, and microbiological data. Detailed information regarding the collected anamnestic and clinical data is provided in the Supplementary method S1. Microbial data (associated infection sites, organisms, and susceptibility) were extracted from the Infectious Diseases Department database. The focus of infection was identified by infectious disease specialists based on detailed physical examinations and confirmation of information in medical records. The primary outcome of this study was the 90-day mortality rate. Secondary outcomes included the risk factors for PB and mortality.

This study was conducted in accordance with the Declaration of Helsinki and approved by the Human Ethical and Clinical Trial Committee of Tohoku University Hospital (2018–1-736). The requirement for patient consent was waived due to the retrospective nature of the study.

### 2.2 Definitions and outcomes

The definitions of BC collection, BSI, PB, follow-up BC, PB duration, PB clearance, contamination, neutropenia, intravascular devices, source control, and adequacy of antimicrobial therapy were adopted from our previous report (13). Bacteremia was classified as nosocomially acquired, healthcare-related, or community-acquired by applying previously described criteria (17). The quick Sequential Organ Failure Assessment (qSOFA) score was calculated based on previously established definitions (18). Comorbidities were identified using the Charlson Comorbidity Scoring System (19). The 90-day mortality rate was defined as death within 90 days of the first BC collection.

The histological type and tumor grading were assessed in accordance with the International Classification of Diseases for Oncology, third edition (20), third edition first revision (21), and third edition second revision (22). Tumor staging was performed based on the Union for International Cancer Control TNM classification, including the sixth (2002) (23), seventh (2010) (24), and eighth (2017) editions (25). Immunohistochemical analysis of p16 expression in patients with oropharyngeal cancer served as an indicator of human papillomavirus status. Oral and laryngopharyngeal mucositis and dermatitis were classified according to the Common Terminology Criteria for Adverse Events Version 4.0 (26).

Details pertaining to the methodology for identifying microorganisms, measuring antimicrobial susceptibility, and performing statistical analyses are provided in the Supplementary methods S2, S4.

## 3 Results

### 3.1 Time series analysis of morbidity and mortality rates in PB and TB hospitalizations

**Figure 1:**
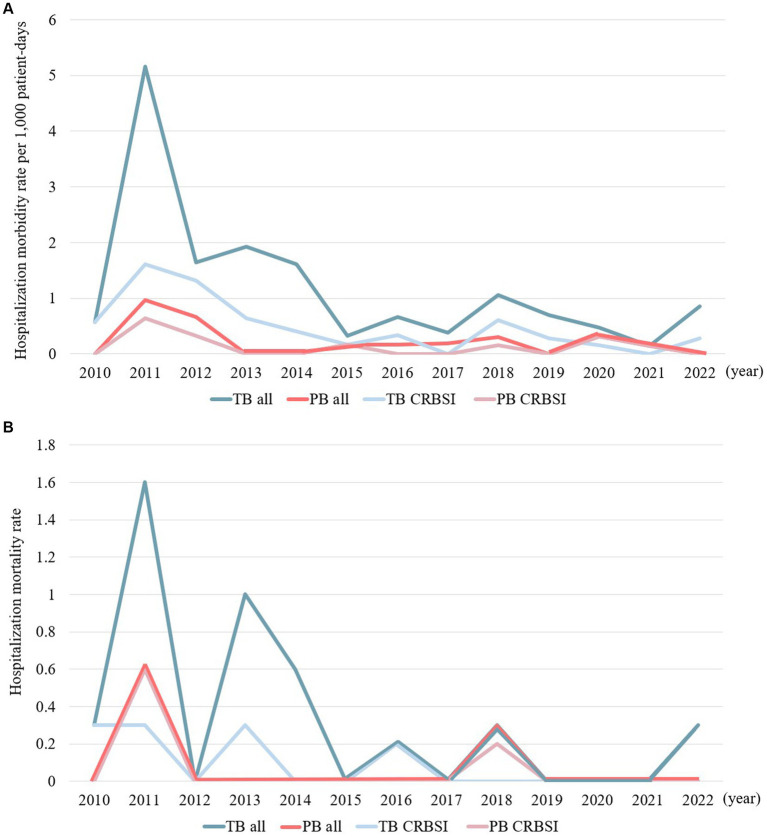
**(A)** Temporal trends in the hospitalization morbidity rates per 1,000 patient days in cases of persistent and transient bacteremia. **(B)** Temporal trends in hospitalization mortality rates in cases of persistent and transient bacteremia.

Figures 1A,B illustrate the temporal changes in hospitalization morbidity rates per 1,000 patient days and mortality rates for the PB and TB groups. Hospitalization morbidity rates per 1,000 patient days and mortality rates exhibited similar trends over time, with both groups peaking in 2011. In more recent years, an increased trend was observed in hospitalization morbidity and mortality rates for TB cases. Catheter-related bloodstream infection (CRBSI) accounted for a significant proportion of the TB and PB groups, serving as a focal point of infection.

### 3.2 Comparing the clinical features of patients with HNC presenting with PB and TB

The clinical characteristics of PB and TB in patients with HNC are shown in Table 1. During the study period, there were 97 cases of positive BCs in patients with HNC; of these, 14 (14%) exhibited PB. Significantly fewer cases of inappropriate antimicrobial use were observed in the PB group (0 cases; 0%) than in the TB group (23 cases; 28%; OR, 0; CI not applicable). In contrast, the number of cases with insufficient source control measures was significantly higher in the PB group than in the TB group (odds ratio [OR], 6.2; 95% confidence interval [CI] 1.4–27.1, *p* = 0.023).

**Table 1:** Differences in clinical characteristics between the persistent and transient bacteremia groups in patients with head and neck cancer.

Characteristic	PB group (*n* = 14)	TB group (*n* = 83)	Odds ratio [95% CI]	*p*-value
**Demography**				
Sex (male, %)	13 (93%)	59 (71%)	5.3 [0.7–42.7]	
Age, years, median (IQR)	56.0 (55.5–56.5)	60.5 (55.8–65.3)		
**Underlying medical conditions**				
Alcohol consumption				
Active	10 (71%)	35 (42%)	3.4 [1–11.8]	
Former	2 (14%)	16 (19%)	0.7 [0.1–3.4]	
Never	2 (14%)	32 (39%)	0.3 [0.1–1.3]	
Smoking				
Active	8 (57%)	24 (29%)	3.3 [1–10.5]	
Former	4 (29%)	35 (42%)	0.5 [0.2–1.9]	
Never	2 (14%)	24 (29%)	0.4 [0.1–2]	
Duplicate cancer	2 (14%)	19 (23%)	0.6 [0.1–2.7]	
Cancer treatment history	3 (21%)	13 (16%)	1.5 [0.4–6]	
Previous antimicrobial therapy	10 (71%)	57 (69%)	1.1 [0.3–4]	
Previous hospital admission	3 (21%)	11 (13%)	1.8 [0.4–7.4]	
Previous corticosteroid therapy	1 (7%)	17 (21%)	0.3 [0–2.4]	
Presence of intensive care unit	0 (0%)	5 (6%)	0	
Shock at presentation	1 (7%)	14 (17%)	0.4 [0–3.1]	
Mucositis, maximum grade (v4)				
Grade 0–2	10 (71%)	71 (86%)	0.4 [0.1–1.6]	
Grade 3–4	3 (21%)	7 (8%)	3 [0.7–13.2]	
Unknown	0 (0%)	5 (6%)	0	
**Vital signs**				
BMI, kg/m^2^, median (IQR)	18.8 (17.3–20.3)	19.8 (17.2–22.3)		
Body temperature, °C, median (IQR)	37.7 (36.7–38.8)	38.0 (37.0–39.1) (n = 68)		
**Laboratory markers**				
White blood cell count, 10^9^/L, median (IQR)	7,200 (4,700–13,900)	8,100 (4,500–11,600)		
Neutrophil cell count, 10^9^/L, median (IQR)	5,100 (3,300–6,600) (*n* = 10)	6,300 (3,300–9,800) (*n* = 71)		
C-reactive protein, mg/dL, median (IQR)	8.5 (5.0–13.6)	7.9 (3.5–18.1)		
Albumin, g/dL, median (IQR)	2.5 (2.3–3.1)	2.7 (2.4–3.1)		
Neutropenia	0 (0%)	5 (6%)	0	
Hypoalbuminemia (<30 g/L)	10 (71%)	57 (69%)	1.1 [0.3–4]	
**Overall condition status score screening tool**				
qSOFA				
0,1	13 (93%)	65 (78%)	3.6 [0.4–29.4]	
>2	1 (7%)	11 (13%)	0.5 [0.1–4.2]	
Beyond evaluation	0 (0%)	7 (8%)	0	
Charlson comorbidity index	10.0 (7.0–11.8)	10.0 (7.0–11.0)		
**Site of acquisition**				
Nosocomial	11 (79%)	75 (90%)	0.4 [0.1–1.7]	
Health care	3 (21%)	7 (8%)	3 [0.7–13.2]	
Community acquired	0 (0%)	1 (1%)	0	
**Health care-associated exposure**				
Intravascular device	9 (64%)	41 (49%)	1.8 [0.6–6]	
Total parenteral nutrition	4 (29%)	21 (25%)	1.2 [0.3–4.2]	
Nasogastric feeding tube	4 (29%)	20 (24%)	1.3 [0.4–4.5]	
Percutaneous gastrostomy	4 (29%)	16 (19%)	1.7 [0.5–6]	
Tracheostomy	3 (21%)	19 (23%)	0.9 [0.2–3.6]	
Invasive mechanical ventilation	0 (0%)	2 (2%)	0	
**Duration of hospital stay, days, median (IQR)**	91.5 (59.5–141.0)	82.0 (46.0–104.5)		
**Duration from admission to blood culture collection, median (IQR)**	51.5 (2.3–78.5)	42.5 (20.0–59.8)		
**Use of antibiotics (inappropriate)**	0 (0%)	23 (28%)	0	0.036
**Source control (inappropriate)**	4 (29%)	5 (6%)	6.2 [1.4–27.1]	0.023
**Site of infection**				
CRBSI	8 (57%)	36 (43%)	1.7 [0.6–5.5]	
Respiratory tract infections	0 (0%)	10 (12%)	0	
Urinary tract infections	0 (0%)	8 (10%)	0	
Biliary tract infections	0 (0%)	1 (1%)	0	
Intra-abdominal infections	0 (0%)	1 (1%)	0	
Thrombophlebitis	2 (14%)	3 (4%)	4.4 [0.7–29.4]	
Pyogenic spondylitis	4 (29%)	2 (2%)	16.2 [2.6–100]	0.004
Septic embolism	0 (0%)	1 (1%)	0	
Skin and soft tissue infections	0 (0%)	2 (2%)	0	
Abscess	0 (0%)	1 (1%)	0	
Infectious endocarditis	1 (7%)	0 (0%)	–	
Mucositis	0 (0%)	1 (1%)	0	
Intraocular candidiasis	0 (0%)	3 (4%)	0	
Lemierre’s syndrome	0 (0%)	1 (1%)	0	
Meningitis	0 (0%)	1 (1%)	0	
Unknown	1 (7%)	22 (27%)	0.2 [0–1.7]	
**Primary site**				
Oral cavity	3 (21%)	22 (27%)	0.8 [0.2–3]	
Oropharynx				
p16, positive	3 (21%)	0 (0%)	–	0.003
p16, negative	0 (0%)	3 (4%)	0	
p16, unknown	0 (0%)	7 (8%)	0	
Hypopharynx	2 (14%)	25 (30%)	0.4 [0.1–1.9]	
Larynx	3 (21%)	10 (12%)	2 [0.5–8.4]	
Nasal cavity and paranasal sinus	2 (14%)	8 (10%)	1.6 [0.3–8.3]	
External auditory canal	0 (0%)	3 (4%)	0	
Salivary grand	0 (0%)	1 (1%)	0	
Unknown primary	1 (7%)	1 (1%)	6.3 [0.4–107.2]	
Others	0 (0%)	3 (4%)	0	
**Clinical stage**				
Stage I–II	3 (21%)	15 (18%)	1.2 [0.3–5]	
Stage III–IV	9 (64%)	62 (75%)	0.6 [0.2–2]	
Beyond evaluation	2 (14%)	6 (7%)	2.1 [0.4–11.8]	
**Treatment of cancer**				
Surgical treatment	0 (0%)	17 (21%)	0	
Chemotherapy				
FP	0 (0%)	2 (2%)	0	
TPF	0 (0%)	4 (5%)	0	
Adriamycin	1 (7%)	0 (0%)	–	
Other regimens	0 (0%)	2 (2%)	0	
Radiotherapy	1 (7%)	6 (7%)	1 [0.1–8.9]	
Chemoradiotherapy				
CDDP-RT	1 (7%)	12 (15%)	0.5 [0.1–3.8]	
DOC-RT	0 (0%)	2 (2%)	0	
FP-RT	0 (0%)	1 (1%)	0	
TPF-RT	1 (7%)	4 (5%)	1.5 [0.2–14.7]	
DC-RT	1 (7%)	1 (1%)	6.3 [0.4–107.2]	
Biotherapy				
Cmab-FP	2 (14%)	3 (4%)	4.4 [0.7–29.4]	
Cmab-RT	1 (7%)	1 (1%)	6.3 [0.4–107.2]	
Nivolumab	1 (7%)	1 (1%)	6.3 [0.4–107.2]	
Under palliative care/Treatment interest	5 (36%)	27 (33%)	1.2 [0.4–3.8]	
**Mortality**				
90-day mortality	3 (21%)	8 (10%)	2.6 [0.6–11.1]	

Data are presented as numbers (%) unless indicated otherwise. Continuous variables were analyzed using the Mann–Whitney *U*-test, and categorical variables were analyzed using Fisher’s exact test. *p*-values are listed only for those values that showed significant differences. Prior antimicrobial therapy was defined as the administration of any systemic antibiotic for 48 h in the preceding 1 month. Hospitalization history was defined as any hospitalization in the 3 months preceding the onset of bloodstream infection. Current corticosteroid therapy was recorded when a patient was receiving corticosteroids at the time of the episode of bacteremia or in the previous month. Intensive care unit stay history was defined as any intensive care unit stay in the 1 month preceding the onset of bloodstream infection. Shock was defined as a systolic pressure of 90 mmHg that was unresponsive to fluid treatment or required vasoactive drug therapy. Blood tests were performed on the same day as the blood culture collection. If they were not performed on the same day, the most recent blood test results were adopted. Intravascular devices include a central line such as a conventional central venous catheter, peripherally inserted central catheter, tunneled central venous catheter, or implanted central venous port. BMI, body mass index; CDDP, cisplatin; CI, confidence interval; Cmab, cetuximab; CRBSI, catheter-related bloodstream infection; DC, docetaxel + carboplatin; DOC, docetaxel; FP, fluorouracil + cisplatin; IQR, interquartile range; PB, persistent bacteremia; qSOFA, quick sequential organ failure assessment; TB, transient bacteremia; RT, radiation therapy; TPF, docetaxel + cisplatin + fluorouracil.

In terms of infection sites, CRBSI was the most common infection in the PB and TB groups (8 cases [57%] and 36 cases [43%], respectively). In the PB group, pyogenic spondylitis (4 cases; 29%) and thrombophlebitis (2 cases; 14%) were the next most prevalent, whereas respiratory (10 cases; 12%) and urinary tract infections (8 cases; 10%) were more frequent in the TB group. The proportion of patients with pyogenic spondylitis was significantly higher in the PB group than in the TB group (OR, 16.2; 95% CI 2.6–100, *p* = 0.004). Additionally, the incidence of thrombophlebitis tended to be higher in the PB group than in the TB group, although it was not statistically significant (OR, 4.4; 95% CI 0.7–29.4).

Regarding the pharmacological treatment regimen, the proportion of biotherapy, including cetuximab + fluorouracil + cisplatin, cetuximab-RT, and nivolumab, showed a tendency to be higher in the PB group than in the TB group, although the difference was not statistically significant (OR, 4.4 [95% CI 0.7–29.4], OR, 6.3 [95% CI 0.4–107.2], and OR, 6.3 [95% CI 0.4–107.2], respectively). When comparing the 90-day mortality rates between the PB and TB groups, the PB group tended to exhibit a higher mortality rate (OR, 2.6; 95% CI 0.6–11.1), although no statistically significant difference was observed. Additionally, the group in which PB clearance was not achieved tended to have a higher mortality rate than the group in which PB clearance was achieved (OR, 9; 95% CI 0.5–155.2).

### 3.3 Microbiology

**Figure 2:**
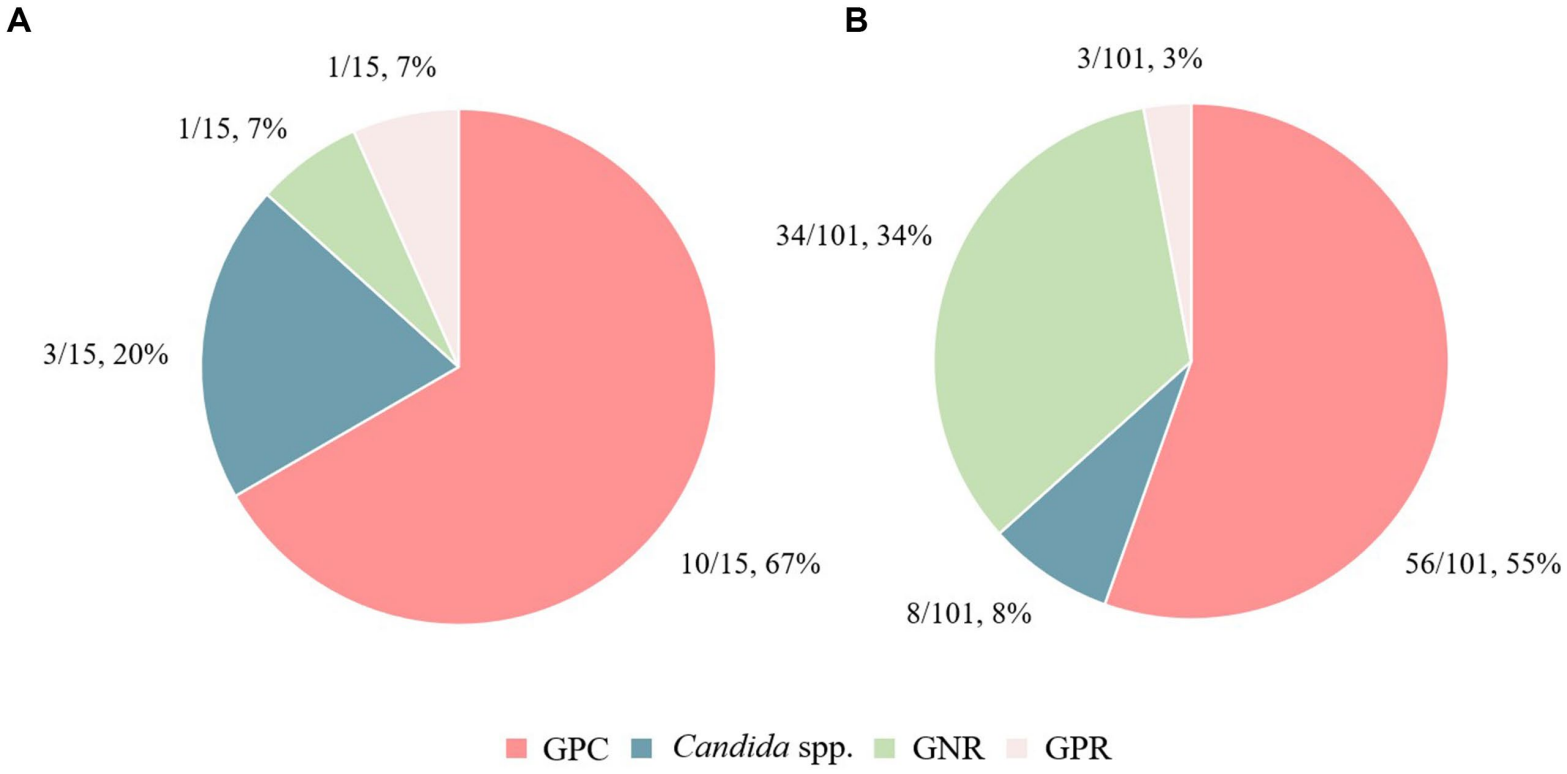
Differences in causative bacterial species between the persistent bacteremia and transient bacteremia groups. The causative bacterial species in **(A)** the persistent bacteremia group and **(B)** the transient bacteremia group are shown. GPC, Gram-positive cocci; GPR, Gram-positive rods; GNR, Gram-negative rods.

Regarding the causative microorganisms of PB and TB in patients with HNC, an overview of the bacterial species based on Gram staining is shown in Figure 2. Detailed bacterial genera and species names are presented in Supplementary Tables S2, S3, highlighting the variation in bacterial species between PB and TB cases, as well as between instances of mortality and survival. GPC tended to occur in a higher proportion of patients in the PB group than in the TB group (67% vs. 55%; OR, 1.6 [95% CI 0.5–5]). Conversely, there was a significantly lower proportion of GNR in the PB group than in the TB group (7% vs. 34%; OR, 0.1 [95% CI 0–1.1], *p* = 0.037). In the PB group, the most frequently identified causative pathogen was methicillin-resistant *S. aureus* (MRSA; 33%), followed by methicillin-susceptible *S. aureus* (MSSA) and *Candida parapsilosis* (13%). In contrast, the most frequently identified causative pathogen in the TB group was *Staphylococcus epidermidis* (20%), followed by MRSA (13%), MSSA (9%), and *Pseudomonas aeruginosa* (7%).

### 3.4 Factors independently associated with PB and mortality in patients with BSI and HNC

Table 2 presents the multivariate logistic regression analysis results. The factor independently associated with PB in patients with HNC was pyogenic spondylitis (adjusted odds ratio [aOR], 22.1; 95% CI 2–239.2], *p* = 0.011). Factors independently associated with mortality in patients with HNC were serum albumin levels (aOR, 0.1; 95% CI 0–0.8, *p* = 0.023) and clinical stages I–II (aOR, 12.9; 95% CI 1.8–93.4, *p* = 0.011).

**Table 2:** Risk factors of persistent bacteremia and mortality in patients with head and neck cancer.

Risk factor	Univariate		Multivariate			
			Fully adjusted		Mutually adjusted	
	aOR [95% CI]	*p*-value	aOR [95% CI]	*p*-value	aOR [95% CI]	*p*-value
**Persistent bacteremia**						
Sex	5.3 [0.7–42.7]	0.106	0.4 [0–3.9]	0.427		
Age	–	0.369	1 [0.9–1]	0.331		
Use of antibiotics (inappropriate)	0	0.036	0 [0–0]	0.998	0 [0–0]	0.999
Source control (inappropriate)	6.2 [1.4–27.1]	0.023	2.5 [0.3–19.3]	0.380		
Pyogenic spondylitis	16.2 [2.6–100]	0.004	18.6 [1.4–238.8]	0.025*	22.1 [2–239.2]	0.011*
Oropharynx, p16, positive	–	0.003	0 [0–0]	0.999	0 [0–0]	0.999
**Mortality**						
Sex	0.9 [0.2–3.8]	1.000	8.5 [0.3–275]	0.227		
Age		0.561	0.9 [0.8–1]	0.127		
Previous antibiotic therapy	0.1 [0–0.5]	0.003	0.5 [0–4.9]	0.527		
Albumin	–	<0.001	0.1[0–1.1]	0.055	0.1 [0–0.8]	0.023*
Hypoalbuminemia	–	0.016	0 [0–0]	0.997	0 [0–0]	0.998
qSOFA, 0,1	0.2 [0.1–0.9]	0.037	0 [0–1.8]	0.088		
qSOFA, >2	5.6 [1.3–23.2]	0.028	0 [0–5.1]	0.191		
Duration of hospital stay	–	0.023	1 [0.9–1]	0.092	1 [0.9–1]	0.061
Oropharynx, p16, unknown	7.7 [1.5–40.6]	0.030	28 [0.8–926.9]	0.062	7.2 [0.9–57.4]	0.061
Clinical stage I–II	4.7 [1.2–17.6]	0.029	16 [1.3–201.6]	0.015*	12.9 [1.8–93.4]	0.011*
Under palliative care/Treatment interest	4.3 [1.1–15.9]	0.037	13.7 [0.6–303.7]	0.098		

The multivariate analysis included all variables that showed statistical significance in the single-variable analysis, along with sex and age, resulting in a fully adjusted report. The term “mutually adjusted” describes the outcomes derived from applying the stepwise method to the ultimate selection of variables. aOR, adjusted odds ratio; CI, confidence interval; qSOFA, quick sequential organ failure assessment.

## 4 Discussion

### 4.1 Temporal changes in hospitalization morbidity rates per 1,000 patient days and mortality rates

The hospitalization morbidity rate per 1,000 patient days and hospitalization mortality rate both peaked in 2011. Furthermore, the hospitalization morbidity and mortality rates in the TB group has exhibited an increasing trend since 2022. Since 2011, the medical team managing HNC has actively implemented infection control measures, specifically by increasing opportunities for BC collection and ensuring the collection of two sets of BCs (Supplementary Figure S1). Additionally, until mid-2011, the primary chemotherapeutic regime was docetaxel + cisplatin + fluorouracil, resulting in a high incidence of febrile neutropenia due to hematologic toxicity. These factors may have contributed significantly to the notable increase in hospitalization morbidity rate per 1,000 patient days and hospitalization mortality rate in 2011. More recently, there has been a renewed increase in the hospitalization morbidity and mortality rates in the TB group in our study. It is imperative to monitor infection control measures and treatment management within hospital wards to prevent further increases in the mortality rate of patients with BSIs and HNC.

### 4.2 CRBSI

Recent technological advancements in the healthcare industry have improved surgical techniques, including those used for treating HNC. In the field of chemotherapy, molecular targeted therapy and immunotherapy have emerged alongside conventional treatment regimens (27, 28). Despite the presumed improvement in hygiene management during patient care, the number of CRBSI cases in our department remains high. In the Department of Otolaryngology and Head and Neck Surgery, laryngoscopy is frequently performed during hospitalization to monitor the treatment progress and airway management. Additionally, procedures such as suction with sputum dispersion and postoperative wound care are often performed within the examination units of the ward. In patients with HNC, central venous catheters or ports are frequently inserted for chemotherapy and nutritional supplementation. In these patients, there is a potential risk of contracting CRBSI, especially during techniques involving aerosol dispersion, where droplets may adhere to the catheter surroundings or be transmitted through contact with healthcare personnel. Although our department generally follows the CRBSI guidelines (29), infection prevention strategies focused solely on managing the catheter insertion site have limitations. Thus, it is desirable to reduce the environmental bacterial load alongside catheter management. Our hospital’s infection control team regularly inspects various medical and surgical units, providing infection management guidance. Particularly for patients with antimicrobial-resistant bacteria, the infection control team recommend isolated treatment units, frequent cleaning and disinfection of equipment and chairs, and environmental disinfection using ultraviolet irradiation devices (30). In addition to managing the catheter-insertion site, implementing these environmental disinfection measures to lower the environmental bacterial load is crucial to decrease the incidence of infections, including CRBSI, in patients with HNC. Furthermore, fostering collaboration between the infection control team and medical staff to increase infection management awareness, informed by current issues, proves to be an effective approach.

### 4.3 Comparison of the clinical characteristics of patients with HNC presenting with PB and TB

#### 4.3.1 Pyogenic spondylitis

RT induces endothelial damage and vascular narrowing, increasing the risk of delayed wound healing and infection (31). Moreover, chemotherapy can cause microcirculatory disturbances and mucositis (32). In our study, CRT was the most common treatment modality in patients with pyogenic spondylitis, accounting for half of the cases (3/6 cases; 50%). Contrastingly, a previous study reported cases of patients with oropharyngeal cancer who developed inflammation of the vertebral bodies and intervertebral discs after oral surgery (33). Therefore, postoperative patients should be monitored for pyogenic spondylitis, similar to those receiving CRT.

Primarily, pyogenic spondylitis is treated with long-term antimicrobial therapy (34). Surgical intervention, however, is recommended in instances of spinal instability, vertebral destruction, abscess formation in the lumbar muscles, and nerve damage (35, 36). In this study, 33% (2/6 cases) underwent debridement for pyogenic spondylitis, while 67% (4/6 cases) did not. Notably, 75% (3/4 cases) of those without source control progressed to PB. In patients with HNC, their compromised overall condition or nutritional status due to cancer may discourage invasive interventions. Hence, this may be a contributing factor to the high proportion of cases for which source control was not implemented to treat pyogenic spondylitis. Multivariate analysis highlighted pyogenic spondylitis as a PB risk factor. Thus, the timely implementation of source control, when necessary, may contribute to the prevention of progression to PB. Furthermore, close monitoring for PB progression is essential, particularly in cases lacking source control.

#### 4.3.2 Mortality

Various bacterial species such as *S. aureus* (10), *Enterococcus* spp. (37), Gram-negative bacteria (11), and *Candida* spp. (12) are reportedly associated with PB and worsened prognosis, particularly leading to increased mortality rates. Cancer status is considered a risk factor for PB (37), and PB is regarded as a risk factor for increased mortality in patients with cancer (38, 39). In this study, even among patients with HNC, the PB group showed a stronger tendency toward higher mortality compared to that of the TB group. Therefore, in patients with HNC who develop PB, careful consideration should be given to appropriate treatment, active management of the infectious focus, and comprehensive systemic management.

In this study, a tendency toward higher mortality rates was observed in cases for which PB clearance was not confirmed (50%, 2/4 cases) compared to that with cases with confirmed clearance (10%, 1/10 cases; OR, 9 [95% CI, 0.5–155.2]). Our previous study demonstrated a similar trend in cases associated with GPC, GNR, and *Candida* spp., with the groups without PB clearance having a higher mortality rate than that of those with clearance (13). The results of this study suggest that even in cases with PB in patients with HNC, confirming its clearance may improve patient prognosis.

#### 4.3.3 Microbial evaluation of PB and TB in patients with HNC

In a retrospective study at our university hospital, 13% of BC-positive patients exhibited PB, 53% of which were caused by GPC (13). Among patients with HNC, the PB incidence rate was approximately 14%, aligning closely with the general hospital population’s rate. Notably, 67% of these PB cases were due to GPC, a marginally higher proportion compared to the general hospital population. Regarding the characteristics of causative pathogens in BSI owing to differences in cancer types, GNRs are predominantly isolated in BSI among patients with cancers other than HNCs (38). Contrastingly, GPC are predominantly isolated in BSI among patients with HNC (16). In our study on bacteremia in patients with HNC, GPC also emerged as the most frequent pathogen (13). Integrating these findings with our current study results reveals distinct characteristics in the causative pathogens of BSI, which vary based on differences in cancer types. Despite the variation, GPC remain an important causative agent, even in patients with HNC.

### 4.4 Factors independently linked to mortality in patients with BSI and HNC

Malnutrition or enhanced vascular permeability can lead to low serum albumin levels, which are linked to heightened infection and BSI risks (16). Patients with esophageal cancer with low pretreatment serum albumin levels reportedly have a higher incidence of complications, such as BSIs, respiratory failure, arrhythmias, and heart failure (40). Furthermore, patients with low pretreatment serum albumin levels had higher postoperative mortality rates, and pretreatment serum albumin levels have been identified as predictors of survival (40). In patients with HNC, low albumin levels are also a BSI risk factor, escalating both early and overall mortality rates (16). Our multivariate analysis identified low albumin levels as a mortality risk factor. Thus, in patients with HNC, serum albumin levels are crucial prognostic factors and valuable for prognostic assessment alongside other clinical metrics.

Patients in clinical stages I and II of HNC exhibited a higher mortality risk compared to those in clinical stages III and IV. However, since the number of cases in clinical stages I and II in this study was small (*n* = 18 cases), the results may have been influenced by sample size bias (Supplementary Table S4).

In conclusion, to the best of our knowledge, this is the first study to investigate clinical features of PB in patients with HNC. Although this study was a single-center retrospective investigation with limitations in generalizability, it was conducted over a long period of 15 years, making the results significant. The key findings are summarized as follows: (1) Peaks in hospitalization morbidity rates per 1,000 patient days and mortality rates in the PB and TB groups were observed in 2011. An increasing trend has recently been observed in hospitalization morbidity and mortality rates for the TB group. (2) CRBSI was the main infectious focus in both groups, with a consistently high contribution to BSI. (3) Patients with HNC with pyogenic spondylitis had an elevated PB risk, particularly without source control. (4) MRSA-related BSI in patients with HNC led more often to PB and higher mortality than did MSSA-related BSI. (5) Pyogenic spondylitis increased PB risk, while hypoalbuminemia increased mortality in patients with HNC. (6) PB had a higher mortality rate than did TB, thereby underscoring the need for PB clearance to improve prognosis.

## Data availability statement

The original contributions presented in the study are included in the article/Supplementary material, further inquiries can be directed to the corresponding author/s.

## Ethics statement

The studies involving humans were approved by the Human Ethical and Clinical Trial Committee of Tohoku University Hospital. The studies were conducted in accordance with the local legislation and institutional requirements. The ethics committee/institutional review board waived the requirement of written informed consent for participation from the participants or the participants’ legal guardians/next of kin because the requirement for patient consent was waived due to the retrospective nature of the study.

## Author contributions

SK: Writing – review & editing, Writing – original draft, Methodology, Investigation, Formal analysis, Data curation, Conceptualization. HK: Writing – review & editing, Investigation. RIs: Writing – review & editing, Data curation. MK: Writing – review & editing, Data curation. RIk: Writing – review & editing, Funding acquisition. KH: Writing – review & editing. RK: Writing – review & editing. AO: Writing – review & editing. YK: Funding acquisition, Writing – review & editing.
