# Supplementary material for: Clinical characteristics and outcomes of persistent bacteremia in patients with head and neck cancer in a tertiary care hospital
Source: Front Med (Lausanne). 2024 Jun 19;11:1406983. doi: 10.3389/fmed.2024.1406983 (PMC11232632; doi:10.3389/fmed.2024.1406983)
Supplement: Supplementary file 1 [file Data_Sheet_1.docx]

Supplementary Material

# Supplementary Method 1

## Content of the collected anamnestic and clinical data

The following anamnestic and clinical data were obtained from medical records: demographic information (sex, age, and underlying medical conditions), alcohol consumption, smoking, presence of duplicate cancer, cancer treatment history, previous antimicrobial therapy, previous hospital admission, previous corticosteroid therapy, presence of intensive care unit stay and shock at presentation, presence of mucositis, vital signs (body mass index and body temperature), laboratory markers (levels of serum white blood cells, neutrophils, C-reactive protein, and albumin), overall condition status score screening tool (quick sequential organ failure assessment score, Charlson comorbidity index), site of acquisition, health care-associated exposure (presence of intravascular device, presence of total parenteral nutrition, presence of nasogastric feeding tube, presence of percutaneous gastrostomy, presence of tracheotomy, presence of mechanical ventilation), duration of hospitalization, duration from admission to blood culture collection, appropriateness of antimicrobial use, performance of source control, site of infections, tumor-related aspects (primary site, clinical stage, treatment of cancer), and 90-day mortality.

# Supplementary Method 2

## Microbial identification

Bacterial strain identification was conducted using the VITEK 2 system (bioMérieux, St. Louis, MO) before August 2010. From March 2013 onwards, the VITEK MS system (bioMérieux, Marcy-l’Étoile, France) was utilized for this purpose.

## Antimicrobial susceptibility testing

Before February 2013, antimicrobial susceptibility testing was performed using the VITEK 2 system (bioMérieux, St. Louis, MO). For fungi and *Haemophilus influenzae* susceptibility testing, RAISUS (Nissui Pharma, Tokyo, Japan) was employed. From March 2013, the Walk Away 96 Plus system (Siemens Healthcare Diagnostics, Deerfield, IL, USA) was employed, and RAISUS S4 (Nissui Pharma, Tokyo, Japan) has been utilized since December 2020.

The minimum inhibitory concentrations and breakpoints of each bacterium were evaluated according to the Clinical Laboratory Standards Institute guidelines versions M100-S22 (2013–2017) (1) and M100-S26 (2018–2022) (2).

# Supplementary Method 3

## Calculation method for hospitalization morbidity rates per 1,000 patient days and hospitalization mortality rates in cases of persistent and transient bacteremia

The hospitalization morbidity rates per 1,000 days in cases of persistent and transient bacteremia were calculated by dividing the number of relevant hospitalization cases for each group per year by the total number of patients hospitalized in the Department of Otolaryngology, Head and Neck Surgery during the same period; this value was then multiplied by 1,000. The hospitalization mortality rates in cases of persistent and transient bacteremia were calculated by dividing the number of deaths for each group per year by the total number of patients hospitalized in the Department of Otolaryngology, Head and Neck Surgery, during the same period and then multiplying by 100.

# Supplementary Method 4

**4.1 Statistical analysis**

The findings of this study were expressed as the median value with a 95% confidence interval or as a proportion of the total number of patients or isolates. The Mann–Whitney U-test was used to compare the averages of continuous variables, and Fisher’s exact test was used to compare the proportions of categorical variables between the two groups. We performed multivariate logistic regression analysis to identify the risk factors for persistent bacteremia (PB) and 90-day mortality. The multivariate conditional logistic regression analysis included all statistically significant variables in the univariate analysis, including sex and age. The final variable selection was performed using the stepwise method. All analyses were performed using JMP Pro 17 statistical analysis software (SAS Institute, 2022, Cary, NC, USA). The differences were considered significant at a corrected *p* < 0.05.

# Supplementary Discussion

## Site of affliction in pyogenic spondylitis

The most common sites of infection for pyogenic spondylitis are the lumbar spine (45–50%), followed by the thoracic spine (35%), cervical spine (3–20%), and sacral region, with the lumbar and thoracic spines accounting for most of the cases (3). However, patients who have undergone radiation therapy for head and neck cancer (HNC) occasionally develop pyogenic spondylitis in the cervical spine (4-6). This is attributed to radiation-induced ulceration of the mucosa near the cervical spine, leading to direct bacterial infection or radiation-induced bone necrosis, with subsequent infection spreading through the bloodstream (6). Our study found that pyogenic spondylitis most commonly affected the lumbar spine (4/6 cases, 67%), followed by one case each in the thoracic and cervical spines (17%). This distribution mirrors the typical infection sites in pyogenic spondylitis. In lumbar spine cases, over half resulted from hematogenous infections leading to catheter-related bloodstream infection (CRBSI) or infectious endocarditis (IE). Thus, patients with HNC and lower back pain, especially with concurrent infections, should be actively evaluated for pyogenic spondylitis.

## Comparison of the clinical characteristics of patients with HNC and persistent or transient bacteremia

### Appropriateness of antimicrobial use and source control

The blood culture tests conducted at our facility have been reviewed by an antimicrobial stewardship team (AST) composed primarily of infectious disease specialists, pharmacists, and nurses. In cases where infection was suspected based on the results of blood culture, the AST actively advises the attending physician to identify the infection focus, select the type of antimicrobial agent based on the susceptibility testing results, provide guidance on the dosage and duration of antimicrobial agent use, and implement source control measures. In patients with PB, AST intervention frequency in treatment surpassed that in patients with transient bacteremia (TB), leading to increased guidance on antimicrobial agent use. This is a key factor in reducing inappropriate antimicrobial agent use in the PB group compared to the TB group. Prior studies demonstrate that AST intervention shortens the time for selecting suitable antimicrobial agents and reduces hospital stays (7). Thus, for effective PB prevention, ongoing comprehensive AST interventions and proper antimicrobial agent use are essential.

The trend in source control was opposite to that for antimicrobial agent use, as inappropriate use was more frequently observed in the PB group than in the TB group. In examining the focus of infection in patients with poor source control in the PB group, we identified two cases of pyogenic spondylitis, one of pyogenic spondylitis with IE, and one of CRBSI. The non-implementation of source control in pyogenic spondylitis was affected by several factors, particularly deteriorated health from cancer and nutritional deficits hindering invasive procedures. For CRBSI, difficulties in peripheral access required catheter preservation, preventing catheter replacement. Although the AST typically advises on source control, the final decision rests with the attending physician, considering the patient's health status. This study revealed a higher PB progression rate in pyogenic spondylitis cases with insufficient source control. Thus, it is advisable to prioritize source control in such cases, barring significant health impediments.

### Molecular targeted therapy and immunotherapy

Cetuximab is a monoclonal antibody that targets the epidermal growth factor receptor (EGFR) (8). EGFR, which is present on the cell surface, is necessary for the signaling pathways involved in cell proliferation. In many solid cancers, EGFR is overexpressed and is considered the starting point for signaling cancer cell growth (9,10). Cetuximab inhibits the function of this receptor by binding to EGFR and blocking the signaling pathway for cell proliferation (8). EXTREME therapy, which includes cetuximab (platinum-based drugs + fluorouracil + cetuximab), is currently considered a standard treatment for recurrent and metastatic HNC (11). Recently, a phase II trial targeting patients with advanced HNC, combining cetuximab with cisplatin-based chemoradiotherapy, reported a complete response rate of 71% (12). One of the major adverse events associated with cetuximab is skin-related side effects (e.g., acne-like rash, skin dryness, skin cracking, paronychia); when used in combination with chemotherapy, severe skin reactions can occur (13). Additionally, cetuximab combined with radiation therapy can exacerbate radiation dermatitis severity (14). A randomized phase III trial showed that severe skin reactions from cetuximab increase the risk of bacterial skin infections (13). Notably, neutropenia, especially severe in patients receiving platinum-based chemotherapy, raises infection-related complications (13). Patients with HNC under cetuximab therapy face a 2.3-fold higher infection risk (adjusted odds ratio [aOR], 2.3; 95% confidence interval [CI], 1.5–3.5) (15), with older adult patients experiencing up to a 33% infection rate annually (15). Moreover, catheter-related infections are linked to molecular-targeted therapies like cetuximab (16). This study indicated a significantly higher CRBSI incidence with cetuximab (5/7 cases [71%]) compared to that with other treatments (39/90 cases [43%]); odds ratio (OR), 3.2; 95% CI, 0.6–17.8.

Nivolumab, like pembrolizumab, is a human immunoglobulin G4 monoclonal antibody that inhibits programmed cell death-1 activity (17). This drug inhibits the binding of programmed cell death-1 to its ligand, enhancing the proliferation and activation of cancer antigen-specific T cells, thereby demonstrating anti-tumor effects (18). However, the proliferation and activation of T cells may lead to the loss of their ability to recognize host cells, potentially resulting in immune-related adverse events (19,20). Detailed reports on the relationship between nivolumab and infections in patients with HNC are lacking. Nonetheless, similar to cetuximab, immune-related adverse events such as skin disorders, mucosal disorders, and neutropenia have been reported for nivolumab (21-23). Thus, it can be inferred that patients undergoing nivolumab therapy may present an increased risk of infection associated with skin and mucosal disorders and hematological abnormalities. In conclusion, patients undergoing biological therapies such as cetuximab and nivolumab, should exercise caution regarding various infections resulting from side effects such as skin and mucosal disorders, and the associated risk of bloodstream infections (BSIs) should be considered.

## Microbial evaluation of PB and TB in patients with HNC

The PB group exhibited a higher prevalence of *Staphylococcus aureus* compared to the TB group. *S. aureus*, particularly methicillin-resistant *S. aureus* (MRSA), is closely linked to clinical severity and increased mortality rates. Patients with MRSA bacteremia face a 25% mortality rate within 3 months (24). Moreover, persistent *S. aureus* bacteremia (SAB) leads to extended hospital stays, more complications, and increased 30-day mortality rates (25). The risk of mortality rises by 1.16 times with each additional day of bacteremia (25); doubling the duration from 1–2 to 4 days nearly doubles the 90-day mortality rate from 22% to 39% (26). In this study, PB incidence was higher in MRSA BSI cases than in methicillin-susceptible *S. aureus* (MSSA) ones (5/18 cases, 28% vs. 2/11 cases, 18%; OR, 1.7 [95% CI, 0.3–11]).

In instances where MRSA was the causative agent, the likelihood of mortality was higher than in cases involving MSSA. The mortality rate for patients with MRSA BSI was 17% (3/18 cases), while all patients with MSSA survived, highlighting MRSA's virulence in BSI among individuals with HNC (OR, 0; CI, not applicable). In the PB and TB groups, MRSA mortality rates were 20% (1/5 cases) and 15% (2/13 cases), respectively. Notably, in the TB group, deaths attributed to MRSA involved co-infections with *Staphylococcus epidermidis* and *Citrobacter koseri*. Conversely, 36% (4/11 cases) of surviving patients with MRSA in the TB group had polymicrobial infections. This indicates an elevated risk of adverse outcomes in patients with TB co-infected with additional bacterial species.

Independent risk factors for the development of persistent SAB include retained intravascular devices or foreign objects, metastatic infections (such as IE and bone or joint infections), chronic kidney disease, cirrhosis, diabetes mellitus, community-acquired bacteremia, methicillin resistance, CRBSI, and vancomycin trough levels < 15 mg/L (27). Furthermore, SAB can lead to serious complications, such as metastatic infections, which contribute to the development of persistent or recurrent bacteremia. Metastatic infections reportedly occur in 13–39% of cases associated with SAB (28). In our previous study, 54% of patients with SAB experienced metastatic infections, with abscesses, IE, and intravascular device infections being the most commonly localized sites (29). In the current study of SAB in patients with HNC, 40% (2/5 cases) had concomitant metastatic infections, indicating a high rate of metastatic infections in this subset.

In the PB group, the primary infection focus was CRBSI, affecting 57% (8/14 cases) of cases. Within this subgroup, 88% (7/8 cases) underwent appropriate antimicrobial therapy and source control measures, leading to PB clearance in 71% (5/7 cases). Contrastingly, one case lacking adequate source control resulted in unresolved PB and mortality. Herein, MRSA was identified as the primary pathogen in 33% (3/9 cases) of PB-CRBSI cases in HNC patients. Our prior research on persistent SAB in patients with CRBSI showed that 95% (38/40 cases) who received proper source control, including central venous catheter management, survived, whereas 50% (1/2 cases) without such measures succumbed (25). These findings suggest that in patients with CRBSI and HNCs, effective antimicrobial use and appropriate source control are crucial for enhancing clinical outcomes.

Furthermore, pathogens other than MRSA in PB-CRBSI, coagulase-negative staphylococci (CoNS), such as *S. epidermidis* and *Staphylococcus hominis*, were identified in two cases. In patients with HNC experiencing BSI, catheter retention for nutrition administration is common due to oral intake challenges. This study found that 52% (50/97 cases) retained catheters, with a higher incidence in the PB group than in the TB group (64% [9/14 cases] vs. 49% [41/83 cases]; OR, 1.8 [95% CI, 0.6–6]). Additionally, the side effects of chemotherapy and the debilitating impact of malignancy often lead to immunosuppression in these patients. Our previous study revealed that in immunosuppressed patients or those with metastatic infections, the mortality rate for CoNS-PB is nearly as high as that for *S. aureus* (29). Therefore, in patients with HNC, it is crucial to be aware that even CoNS, which is not typically considered highly pathogenic, can be the causative agent in cases of CRBSI and may lead to sustained bacteremia.

# References

1. Clinical and Laboratory Standards Institute (2012) M100-S22. Performance standards for antimicrobial susceptibility testing; 22th informational supplement. Clinical and Laboratory Standards Institute, Wayne.
2. Clinical and Laboratory Standards Institute (2016) M100-S26. Performance standards for antimicrobial susceptibility testing; 26th informational supplement. Clinical and Laboratory Standards Institute, Wayne
3. Jaramillo-de la Torre JJ, Bohinski RJ, Kuntz Ct. Vertebral osteomyelitis. Neurosurg Clin N Am (2006) 17:339-51. https://doi.org/10.1016/j.nec.2006.05.003
4. Yung CS, Leung DKC, Cheung JPY. The prevalence and impact of cervical spine pathologies in patients with nasopharyngeal carcinoma. Oral Oncol (2019) 90:48-53. https://doi.org/10.1016/j.oraloncology.2019.01.013
5. Khorsandi AS, Su HK, Mourad WF, Urken ML, Persky MS, Lazarus CL, et al. Osteoradionecrosis of the subaxial cervical spine following treatment for head and neck carcinomas. Br J Radiol (2015) 88:20140436. https://doi.org/10.1259/bjr.20140436
6. Karakida K, Uchibori M, Nakanishi Y, Tamura M, Takahashi M, Hoshimoto Y, et al. Pyogenic spondylitis with rapid bone destruction after chemoradiotherapy for tongue cancer: A case report and literature review. Tokai J Exp Clin Med (2020) 45:182-8.
7. O'Donnell JN, Rhodes NJ, Miglis CM, Zembower TR, Qi C, Hoff BM, et al. Impact of early antimicrobial stewardship intervention in patients with positive blood cultures: results from a randomized comparative study. Int J Antimicrob Agents (2022) 59:106490. https://doi.org/10.1016/j.ijantimicag.2021.106490
8. Bonner JA, Harari PM, Giralt J, Azarnia N, Shin DM, Cohen RB, et al. Radiotherapy plus cetuximab for squamous-cell carcinoma of the head and neck. N Engl J Med (2006) 354:567-78. https://doi.org/10.1056/NEJMoa053422
9. Ciardiello F, Tortora G. EGFR antagonists in cancer treatment. N Engl J Med (2008) 358:1160-74. https://doi.org/10.1056/NEJMra0707704
10. Salomon DS, Brandt R, Ciardiello F, Normanno N. Epidermal growth factor-related peptides and their receptors in human malignancies. Crit Rev Oncol Hematol (1995) 19:183-232. https://doi.org/10.1016/1040-8428(94)00144-i
11. Vermorken JB, Mesia R, Rivera F, Remenar E, Kawecki A, Rottey S, et al. Platinum-based chemotherapy plus cetuximab in head and neck cancer. N Engl J Med (2008) 359:1116-27. https://doi.org/10.1056/NEJMoa0802656
12. Merlano M, Russi E, Benasso M, Corvò R, Colantonio I, Vigna-Taglianti R, et al. Cisplatin-based chemoradiation plus cetuximab in locally advanced head and neck cancer: a phase II clinical study. Ann Oncol (2011) 22:712-7. https://doi.org/10.1093/annonc/mdq412.
13. Marín M, Gudiol C, Castet F, Oliva M, Peiró I, Royo-Cebrecos C, et al. Bloodstream infection in patients with head and neck cancer: a major challenge in the cetuximab era. Clin Transl Oncol (2019) 21:187-96. https://doi.org/10.1007/s12094-018-1905-5
14. Ferris RL, Blumenschein G Jr, Fayette J, Guigay J, Colevas AD, Licitra L, et al. Nivolumab for recurrent squamous-cell carcinoma of the head and neck. N Engl J Med (2016) 375:1856-67. https://doi.org/10.1056/NEJMoa1602252
15. Lee CC, Ho HC, Hsiao SH, Huang TT, Lin HY, Li SC, et al. Infectious complications in head and neck cancer patients treated with cetuximab: propensity score and instrumental variable analysis. PLoS One (2012) 7:e50163. https://doi.org/10.1371/journal.pone.0050163
16. Kitaya S, Kanamori H, Baba H, Oshima K, Takei K, Seike I, et al. Clinical and epidemiological characteristics of persistent bacteremia: A decadal observational study. Pathogens (2023) 12:212. https://doi.org/10.3390/pathogens12020212
17. Jin HT, Ahmed R, Okazaki T. Role of PD-1 in regulating T-cell immunity. Curr Top Microbiol Immunol (2011) 350:17-37. https://doi.org/10.1007/82_2010_116
18. Okazaki T, Honjo T. PD-1 and PD-1 ligands: from discovery to clinical application. Int Immunol (2007) 19:813-24. https://doi.org/10.1093/intimm/dxm057
19. Cramer JD, Burtness B, Ferris RL. Immunotherapy for head and neck cancer: Recent advances and future directions. Oral Oncol (2019) 99:104460. https://doi.org/10.1016/j.oraloncology.2019.104460
20. Kadono T. Immune-related adverse events by immune checkpoint inhibitors. Nihon Rinsho Meneki Gakkai Kaishi (2017) 40:83-9 [in Japanese].
21. Osawa T, Inoue S, Umeda M, Hasegawa T, Makino T, Hori A, et al. Predictors of nivolumab-induced skin reactions. Gan To Kagaku Ryoho (2018) 45:1533-5 [in Japanese].
22. DI Cosola M, Spirito F, Saracino P, Caponio VC, Diaz-Flores Garcia V, Madonna G, et al. Oral immune-related adverse events caused by immune checkpoint inhibitors: a retrospective study. Minerva Dent Oral Sci (2022) 71:318-23. https://doi.org/10.23736/S2724-6329.22.04768-4
23. Kalaci E, Ürün Y. Nivolumab-induced immune-related neutropenia in a renal cell carcinoma patient. J Oncol Pharm Pract (2023) 29:1797-801. https://doi.org/10.1177/10781552231186748
24. Bai AD, Lo CKL, Komorowski AS, Suresh M, Guo K, Garg A, et al. *Staphylococcus aureus* bacteraemia mortality: a systematic review and meta-analysis. Clin Microbiol Infect (2022) 28:1076-84. https://doi.org/10.1016/j.cmi.2022.03.015
25. Minejima E, Mai N, Bui N, Mert M, Mack WJ, She RC, et al. Defining the breakpoint duration of *Staphylococcus aureus* bacteremia predictive of poor outcomes. Clin Infect Dis (2020) 70:566-73. https://doi.org/10.1093/cid/ciz257
26. Kuehl R, Morata L, Boeing C, Subirana I, Seifert H, Rieg S, et al. Defining persistent *Staphylococcus aureus* bacteraemia: secondary analysis of a prospective cohort study. Lancet Infect Dis (2020) 20:1409-17. https://doi.org/10.1016/S1473-3099(20)30447-3
27. Chong YP, Park SJ, Kim HS, Kim ES, Kim MN, Park KH, et al. Persistent *Staphylococcus aureus* bacteremia: a prospective analysis of risk factors, outcomes, and microbiologic and genotypic characteristics of isolates. Medicine (Baltimore) (2013) 92:98-108. https://doi.org/10.1097/MD.0b013e318289ff1e.
28. Karlowicz MG, Furigay PJ, Croitoru DP, Buescher ES. Central venous catheter removal versus in situ treatment in neonates with coagulase-negative staphylococcal bacteremia. Pediatr Infect Dis J (2002) 21:22–27. https://doi.org/10.1097/00006454-200201000-00005
29. Kitaya S, Kanamori H, Katori Y, Tokuda K. Clinical characteristics and outcomes of persistent Staphylococcal bacteremia in a tertiary care hospital. Antibiotics (Basel) (2023) 12:454. https://doi.org/10.3390/antibiotics12030454

# Supplementary Figures and Tables

**Supplementary Table 1.** Differences in clinical characteristics between the survivor and fatality groups among patients with bacteremia and head and neck cancers.

| Characteristic | Fatality group  (n = 11) | Survivor group  (n = 86) | Odds ratio  [95% CI] | *p*-value |
| --- | --- | --- | --- | --- |
| **Demography** |  |  |  |  |
| Sex (male, %) | 8 (73%) | 64 (74%) | 0.9 [0.2–3.8] |  |
| Age, years, median (IQR) | 66.0 (61.0–76.0) | 70.0 (64.3–75.0) |  |  |
| **Underlying medical conditions** |  |  |  |  |
| Alcohol consumption |  |  |  |  |
| Active | 7 (64%) | 38 (44%) | 2.2 [0.6–8.1] |  |
| Former | 1 (9%) | 17 (20%) | 0.4 [0–3.4] |  |
| Never | 3 (27%) | 31 (36%) | 0.7 [0.2–2.7] |  |
| Smoking |  |  |  |  |
| Active | 5 (46%) | 27 (31%) | 1.8 [0.5–6.5] |  |
| Former | 3 (27%) | 36 (42%) | 0.5 [0.1–2.1] |  |
| Never | 3 (27%) | 23 (27%) | 1 [0.3–4.2] |  |
| Duplicate cancer | 2 (18%) | 19 (22%) | 0.8 [0.2–3.9] |  |
| Cancer treatment history | 3 (27%) | 13 (15%) | 2.1 [0.5–9] |  |
| Previous antimicrobial therapy | 3 (27%) | 64 (74%) | 0.1 [0–0.5] | 0.003 |
| Previous hospital admission | 2 (18%) | 12 (14%) | 1.4 [0.3–7.1] |  |
| Previous corticosteroid therapy | 2 (18%) | 16 (19%) | 1 [0.2–4.9] |  |
| Presence of intensive care unit | 0 (0%) | 5 (6%) | 0 |  |
| Shock at presentation | 1 (9%) | 14 (16%) | 0.5 [0.1–4.3] |  |
| Mucositis, maximum grade (v4) |  |  |  |  |
| Grade 0–2 | 9 (82%) | 72 (84%) | 0.9 [0.2–4.5] |  |
| Grade 3–4 | 1 (9%) | 9 (11%) | 0.9 [0.1–7.5] |  |
| Unknown | 1 (9%) | 4 (5%) | 2.1 [0.2–20.2] |  |
| **Vital signs** |  |  |  |  |
| BMI, kg/m², median (IQR) | 17.7 (15.6–21.9) | 19.8 (17.4–21.9) |  |  |
| Body temperature, ℃, median (IQR) | 37.8 (37.3–38.6) (n = 8) | 37.9 (37.0–39.1) (n = 74) |  |  |
| **Laboratory markers** |  |  |  |  |
| White blood cell count, 10⁹/L, median (IQR) | 10,600 (7,600–15,300) | 7,500 (4,400–11,100) |  |  |
| Neutrophil cell count, 10⁹/L, median (IQR) | 8,700 (5,400–13,400) (n = 9) | 6,100 (3,200–9,600) (n = 72) |  |  |
| C-reactive protein, mg/dL, median (IQR) | 10.5 (9.6–16.0) | 7.1 (3.4–16.3) |  |  |
| Albumin, g/dL, median (IQR) | 2.0 (1.7–2.6) | 2.8 (2.5–3.1) |  | <0.001 |
| Neutropenia | 1 (9%) | 4 (5%) | 2.1 [0.2–20.2] |  |
| Hypoalbuminemia (< 30g/L) | 11 (100%) | 56 (65%) | – | 0.016 |
| **Overall condition status score screening tool** |  |  |  |  |
| qSOFA |  |  |  |  |
| 0,1 | 6 (55%) | 72 (84%) | 0.2 [0.1–0.9] | 0.037 |
| > 2 | 4 (36%) | 8 (9%) | 5.6 [1.3–23.2] | 0.028 |
| Beyond evaluation | 1 (9%) | 6 (7%) | 1.3 [0.1–12.2] |  |
| Charlson comorbidity index | 11.0 (8.0–11.5) | 10.0 (7.0–11.0) |  |  |
| **Site of acquisition** |  |  |  |  |
| Nosocomial | 9 (82%) | 77 (90%) | 0.5 [0.1–2.8] |  |
| Health care | 2 (18%) | 8 (9%) | 2.2 [0.4–11.8] |  |
| Community acquired | 0 (0%) | 1 (1%) | 0 |  |
| **Health care-associated exposure** |  |  |  |  |
| Intravascular device | 7 (64%) | 43 (50%) | 1.8 [0.5–6.4] |  |
| Total parenteral nutrition | 2 (18%) | 23 (27%) | 0.6 [0.1–3] |  |
| Nasogastric feeding tube | 4 (36%) | 20 (23%) | 1.9 [0.5–7.1] |  |
| Percutaneous gastrostomy | 2 (18%) | 18 (21%) | 0.8 [0.2–4.2] |  |
| Tracheostomy | 5 (46%) | 17 (20%) | 3.4 [0.9–12.4] |  |
| Invasive mechanical ventilation | 1 (9%) | 1 (1%) | 8.5 [0.5–146.7] |  |
| **Persistent bacteremia** | 3 (27%) | 11 (13%) | 2.6 [0.6–11.1] |  |
| **Duration of hospital stay, days, median (IQR)** | 53.0 (30.0–80.0) | 89.0 (54.3–112.5) |  | 0.023 |
| **Duration from admission to blood culture collection, median (IQR)** | 24.0 (4.0–63.0) | 44.0 (17.0–60.0) |  |  |
| **Use of antibiotics (inappropriate)** | 1 (9%) | 22 (26%) | 0.3 [0–2.4] |  |
| **Source control (inappropriate)** | 3 (27%) | 6 (7%) | 5 [1–23.9] |  |
| **Site of infection** |  |  |  |  |
| CRBSI | 4 (36%) | 40 (47%) | 0.7 [0.2–2.4] |  |
| Respiratory tract infections | 3 (27%) | 7 (8%) | 4.2 [0.9–19.7] |  |
| Urinary tract infections | 2 (18%) | 6 (7%) | 3 [0.5–16.9] |  |
| Biliary tract infections | 0 (0%) | 1 (1%) | 0 |  |
| Intra-abdominal infections | 1 (9%) | 0 (0%) | – |  |
| Thrombophlebitis | 1 (9%) | 4 (5%) | 2.1 [0.2–20.2] |  |
| Pyogenic spondylitis | 0 (0%) | 6 (7%) | 0 |  |
| Septic embolism | 0 (0%) | 1 (1%) | 0 |  |
| Skin and soft tissue infections | 0 (0%) | 2 (2%) | 0 |  |
| Abscess | 1 (9%) | 0 (0%) | – |  |
| Infectious endocarditis | 0 (0%) | 1 (1%) | 0 |  |
| Mucositis | 0 (0%) | 1 (1%) | 0 |  |
| Intraocular candidiasis | 0 (0%) | 3 (4%) | 0 |  |
| Lemierre's syndrome | 0 (0%) | 1 (1%) | 0 |  |
| Meningitis | 0 (0%) | 1 (1%) | 0 |  |
| Unknown | 1 (9%) | 22 (26%) | 0.3 [0–2.4] |  |
| **Primary site** |  |  |  |  |
| Oral cavity | 2 (18%) | 23 (27%) | 0.6 [0.1–3] |  |
| Oropharynx |  |  |  |  |
| p16, positive | 1 (9%) | 2 (2%) | 4.2 [0.3–50.6] |  |
| p16, negative | 0 (0%) | 3 (4%) | 0 |  |
| p16, unknown | 3 (27%) | 4 (5%) | 7.7 [1.5–40.6] | 0.030 |
| Hypopharynx | 3 (27%) | 24 (28%) | 1 [0.2–4] |  |
| Larynx | 1 (9%) | 12 (14%) | 0.6 [0.1–5.3] |  |
| Nasal cavity and paranasal sinus | 1 (9%) | 9 (11%) | 0.9 [0.1–7.5] |  |
| External auditory canal | 0 (0%) | 3 (4%) | 0 |  |
| Salivary grand | 0 (0%) | 1 (1%) | 0 |  |
| Unknown primary | 0 (0%) | 2 (2%) | 0 |  |
| Others | 0 (0%) | 3 (4%) | 0 |  |
| **Clinical stage** |  |  |  |  |
| Stage I–II | 5 (46%) | 13 (15%) | 4.7 [1.2–17.6] | 0.029 |
| Stage III–IV | 5 (46%) | 66 (77%) | 0.3 [0.1–0.9] |  |
| Beyond evaluation | 1 (9%) | 7 (8%) | 1.1 [0.1–10.1] |  |
| **Treatment of cancer** |  |  |  |  |
| Surgical treatment | 1 (9%) | 16 (19%) | 0.4 [0.1–3.7] |  |
| Chemotherapy |  |  |  |  |
| FP | 0 (0%) | 2 (2%) | 0 |  |
| TPF | 1 (9%) | 3 (4%) | 2.8 [0.3–29.2] |  |
| Adriamycin | 0 (0%) | 1 (1%) | 0 |  |
| Other regimens | 0 (0%) | 2 (2%) | 0 |  |
| Radiotherapy | 2 (18%) | 5 (6%) | 3.6 [0.6–21.3] |  |
| Chemoradiotherapy |  |  |  |  |
| CDDP-RT | 0 (0%) | 13 (15%) | 0 |  |
| DOC-RT | 0 (0%) | 2 (2%) | 0 |  |
| FP-RT | 0 (0%) | 1 (1%) | 0 |  |
| TPF-RT | 0 (0%) | 5 (6%) | 0 |  |
| DC-RT | 0 (0%) | 2 (2%) | 0 |  |
| Biotherapy |  |  |  |  |
| Cmab-FP | 0 (0%) | 5 (6%) | 0 |  |
| Cmab-RT | 0 (0%) | 2 (2%) | 0 |  |
| Nivolumab | 0 (0%) | 2 (2%) | 0 |  |
| Under palliative care/Treatment interest | 7 (64%) | 25 (29%) | 4.3 [1.1–15.9] | 0.037 |

Data are presented as numbers (%) unless indicated otherwise.

Continuous variables were analyzed using the Mann–Whitney U test, and categorical variables were analyzed using Fisher's exact test.

*P*-values are listed only for those values that showed significant differences.

Prior antimicrobial therapy was defined as the administration of any systemic antibiotic for 48 h in the preceding 1 month.

Hospitalization history was defined as any hospitalization in the 3 months preceding the onset of bloodstream infection.

Current corticosteroid therapy was recorded when a patient was receiving corticosteroids at the time of the episode of bacteremia or in the previous month.

Intensive care unit stay history was defined as any intensive care unit stay in the 1 month preceding the onset of bloodstream infection.

Shock was defined as a systolic pressure of 90 mmHg that was unresponsive to fluid treatment or required vasoactive drug therapy.

Blood tests were performed on the same day as blood culture collection. If they were not performed on the same day, the most recent blood test results were adopted.

Intravascular devices include a central line such as a conventional central venous catheter, peripherally inserted central catheter, tunneled central venous catheter, or implanted central venous port.

BMI, body mass index; CDDP, cisplatin; CI, confidence interval; Cmab, cetuximab; CRBSI, catheter-related bloodstream infection; DC, docetaxel + carboplatin; DOC, docetaxel; FP, fluorouracil + cisplatin; IQR, interquartile range; qSOFA, quick sequential organ failure assessment; RT, radiation therapy; TPF, docetaxel + cisplatin + fluorouracil.

**Supplementary Table 2**. Microbiological assessment of persistent and transient bacteremia in patients with head and neck cancer.

| **Causative organism** | Number | Frequency (%) |
| --- | --- | --- |
| **PB group** |  |  |
| **Gram-positive coccus** |  |  |
| methicillin-resistant *Staphylococcus aureus* | 5 | 33 |
| methicillin-susceptible *Staphylococcus aureus* | 2 | 13 |
| *Staphylococcus capitis* | 1 | 7 |
| *Staphylococcus hominis* | 1 | 7 |
| *Staphylococcus epidermidis* | 1 | 7 |
| **Gram-positive rods** |  |  |
| *Bacillus cereus* | 1 | 7 |
| **Gram-negative rods** |  |  |
| *Enterobacter aerogenes* | 1 | 7 |
| **Fungi** |  |  |
| *Candida parapsilosis* | 2 | 13 |
| *Candida lusitaniae* | 1 | 7 |
| **TB group** |  |  |
| **Gram-positive coccus** |  |  |
| *Staphylococcus epidermidis* | 20 | 20 |
| methicillin-resistant *Staphylococcus aureus* | 13 | 13 |
| methicillin-susceptible *Staphylococcus aureus* | 9 | 9 |
| *Staphylococcus hominis* | 4 | 4 |
| *Streptococcus anginosus* | 2 | 2 |
| *Enterococcus faecalis* | 2 | 2 |
| others | 6 | 6 |
| **Gram-positive rods** |  |  |
| *Bacillus cereus* | 1 | 1 |
| *Bacillus sp.* | 1 | 1 |
| *Clostridium perfringens* | 1 | 1 |
| **Gram-negative rods** |  |  |
| *Pseudomonas aeruginosa* | 7 | 7 |
| *Klebsiella pneumoniae* | 6 | 6 |
| *Enterobacter aerogenes* | 5 | 5 |
| *Klebsiella oxytoca* | 3 | 3 |
| *Escherichia coli* | 4 | 3 |
| *Citrobacter koseri* | 2 | 2 |
| *Enterobacter cloacae* | 2 | 2 |
| others | 5 | 5 |
| **Fungi** |  |  |
| *Candida albicans* | 4 | 4 |
| *Candida parapsilosis* | 4 | 4 |

PB, persistent bacteremia; TB, transient bacteremia.

**Supplementary Table 3.** Microbiological assessments in the fatality and survivor groups due to bloodstream infections in patients with head and neck cancers.

| **Causative organisms** | Number | Frequency (%) |
| --- | --- | --- |
| **Fatality group** |  |  |
| **Gram-positive coccus** |  |  |
| methicillin-resistant *Staphylococcus aureus* | 3 | 21 |
| *Staphylococcus epidermidis* | 2 | 14 |
| *Staphylococcus anginosus* | 1 | 7 |
| *Peptostreptococcus micros* | 1 | 7 |
| **Gram-negative rods** |  |  |
| *Klebsiella pneumoniae* | 2 | 14 |
| *Enterobacter aerogenes* | 2 | 14 |
| *Pseudomonas aeruginosa* | 1 | 7 |
| *Citrobacter koseri* | 1 | 7 |
| **Fungi** |  |  |
| *Candida lusitaniae* | 1 | 7 |
| **Survivor group** |  |  |
| **Gram-positive coccus** |  |  |
| *Staphylococcus epidermidis* | 19 | 19 |
| methicillin-resistant *Staphylococcus aureus* | 15 | 15 |
| methicillin-susceptible *Staphylococcus aureus* | 11 | 11 |
| *Staphylococcus hominis* | 5 | 5 |
| *Streptococcus capitis* | 2 | 2 |
| *Enterococcus faecalis* | 2 | 2 |
| others | 5 | 5 |
| **Gram-positive rods** |  |  |
| *Bacillus cereus* | 2 | 2 |
| *Bacillus sp.* | 1 | 1 |
| *Clostridium perfringens* | 1 | 1 |
| **Gram-negative rods** |  |  |
| *Pseudomonas aeruginosa* | 6 | 6 |
| *Klebsiella pneumoniae* | 4 | 4 |
| *Enterobacter aerogenes* | 4 | 4 |
| *Escherichia coli* | 4 | 4 |
| *Klebsiella oxytoca* | 3 | 3 |
| *Enterobacter cloacae* | 2 | 2 |
| others | 6 | 6 |
| **Fungi** |  |  |
| *Candida parapsilosis* | 6 | 6 |
| *Candida albicans* | 4 | 4 |

**Supplementary Table 4.** Comparison of causative bacterial species for persistent bacteremia based on the staging of head and neck cancer.

| Causative organisms | Fatality group (n = 14) | Survivor group (n = 102) | Odds ratio [95% CI] | *p*-value |
| --- | --- | --- | --- | --- |
| All |  |  |  |  |
| GPC | 7 (50%) | 59 (58%) | 0.7 [0.2–2.2] | 0.581 |
| GPR | 0 (0%) | 4 (4%) | 0 | 1.000 |
| GNR | 6 (43%) | 29 (28%) | 1.9 [0.6–5.9] | 0.352 |
| *Candida* spp. | 1 (7%) | 10 (10%) | 0.7 [0.1–6] | 1.000 |
| Stage I–II |  |  |  |  |
| GPC | 0 (0%) | 7 (50%) | 0 | – |
| GPR | 0 (0%) | 1 (7%) | 0 | – |
| GNR | 4 (80%) | 5 (36%) | 7.2 [0.6–83.3] | 0.141 |
| *Candida* spp. | 1 (20%) | 1 (7%) | 3.3 [0.2–64.6] | 0.468 |
| Stage III–IV |  |  |  |  |
| GPC | 5 (71%) | 48 (59%) | 1.7 [0.3–9.4] | 0.698 |
| GPR | 0 (0%) | 3 (4%) | 0 | 1.000 |
| GNR | 2 (29%) | 22 (27%) | 1.1 [0.2–5.9] | 1.000 |
| *Candida* spp. | 0 (0%) | 8 (10%) | 0 | 1.000 |
| Beyond evaluation |  |  |  |  |
| GPC | 2 (100%) | 4 (57%) | – | 0.500 |
| GPR | 0 (0%) | 0 (0%) | – | – |
| GNR | 0 (0%) | 2 (29%) | 0 | 1.000 |
| *Candida* spp. | 0 (0%) | 1 (14%) | 0 | 1.000 |

Data are presented as numbers (%) unless indicated otherwise.

We analyzed categorical variables using Fisher's exact test.

CI, confidence interval; GNR, Gram-negative rods; GPC, Gram-positive coccus; GPR, Gram-positive rods.


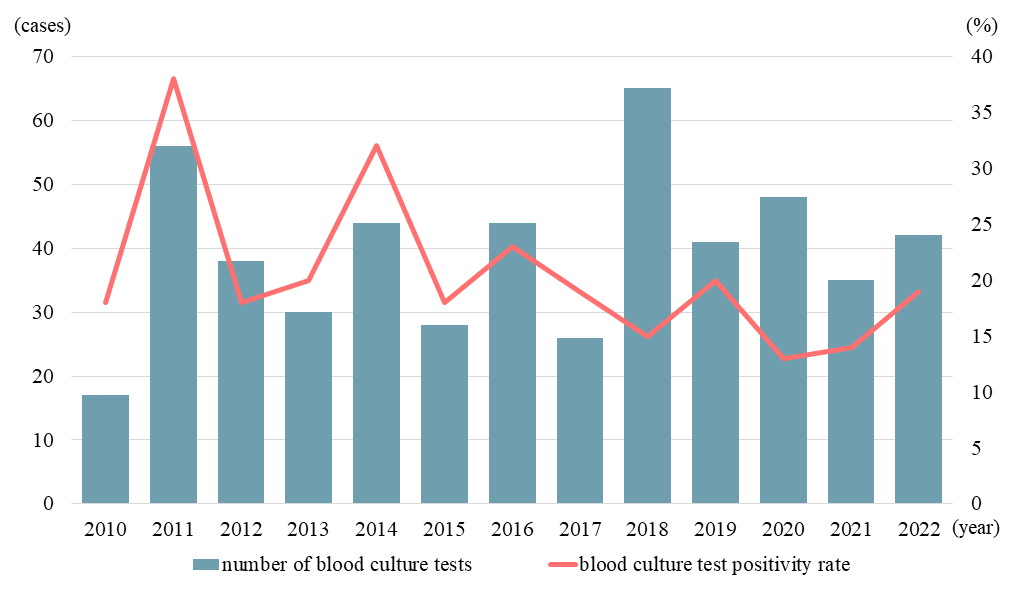


**Supplementary Figure 1.** Temporal changes in the number of blood culture tests conducted and the positivity rate of blood culture tests.

The temporal changes in the number of blood culture tests conducted and the positivity rate of blood culture tests performed during the study period at our Otolaryngology-Head and Neck Surgery department are depicted.
